# Supplementary figures and images for: The super repertoire of type IV effectors in the pangenome of Ehrlichia spp. provides insights into host-specificity and pathogenesis
Source: PLoS Comput Biol. 2021 Jul 12;17(7):e1008788. doi: 10.1371/journal.pcbi.1008788 (PMC8274917; doi:10.1371/journal.pcbi.1008788)

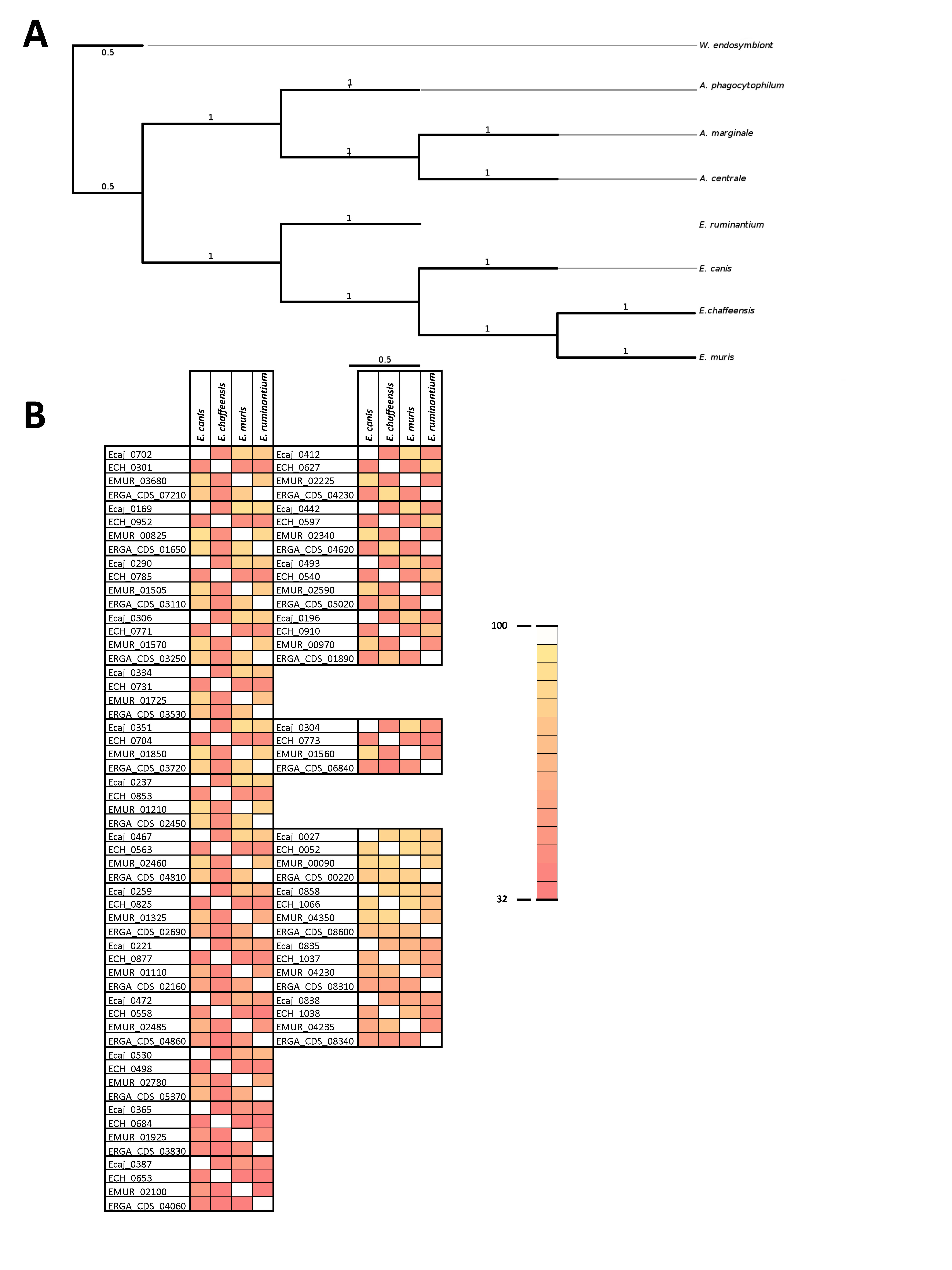

Supplement: S1 Fig — S1A. A maximum-likelihood tree of 4 Ehrlichia species, 3 Anaplasma species and W. endosymbiont of D. melanogaster (out group) was reconstructed on the basis of concatenated nucleic acid alignments of pT4Es shared by all species (core effectome) with 100 bootstrap resamplings. S1B. The identity percentage was calculated for each effector ortholog group (EOG) of the Ehrlichia core effectome, and is represented by a heat map. The colour gradient represents the identity between effectors (pale colours mean high similarity). (TIFF) [file pcbi.1008788.s001.tiff]

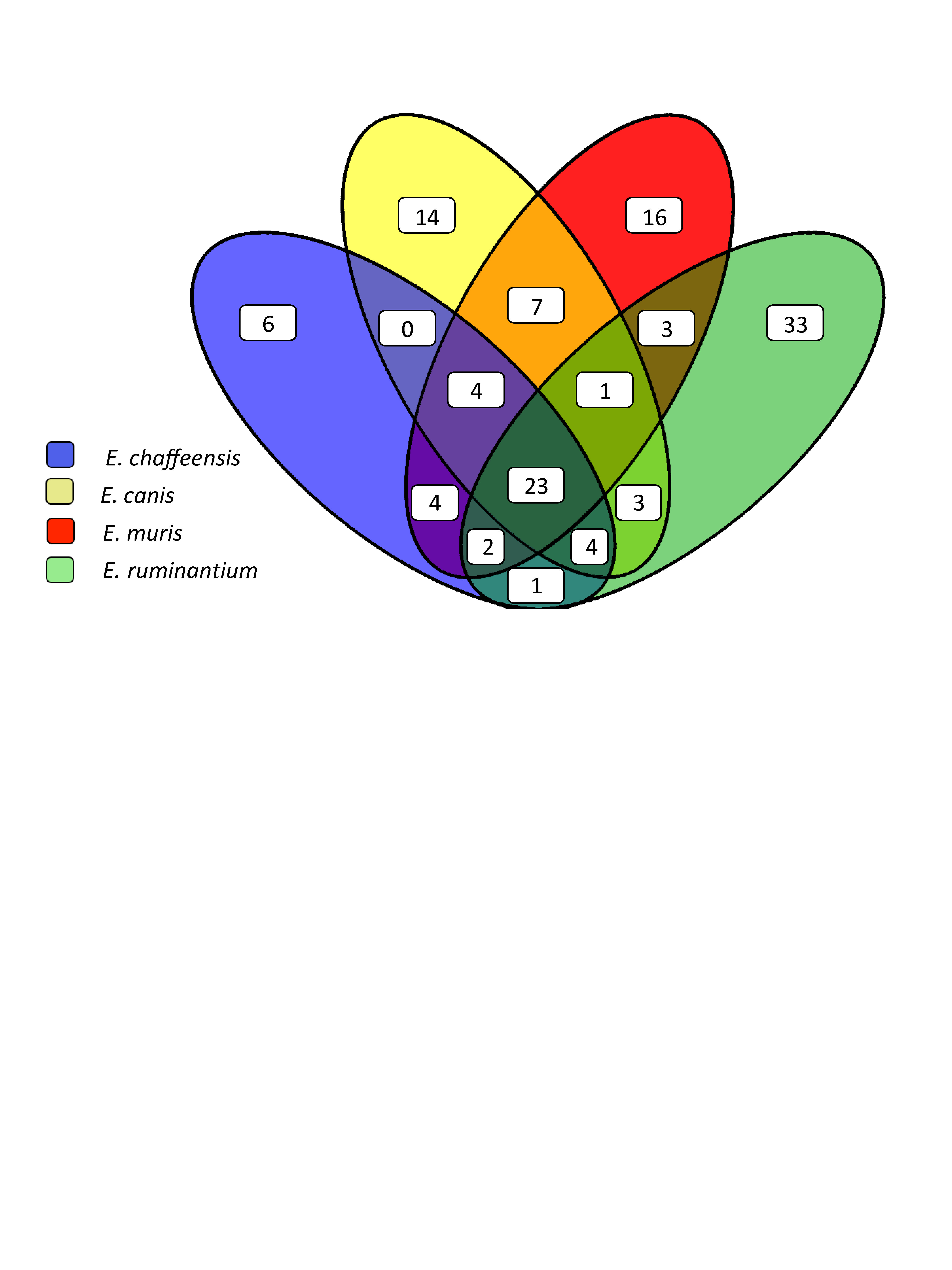

Supplement: S2 Fig — Predicted T4 effectomes of four Ehrlichia species compared with S4TE-CG and PanOCT to find homologous proteins in each species. Results are plotted on a Venn diagram and a number indicates the occurrence of predicted effectors is each intersection. (TIFF) [file pcbi.1008788.s002.tiff]

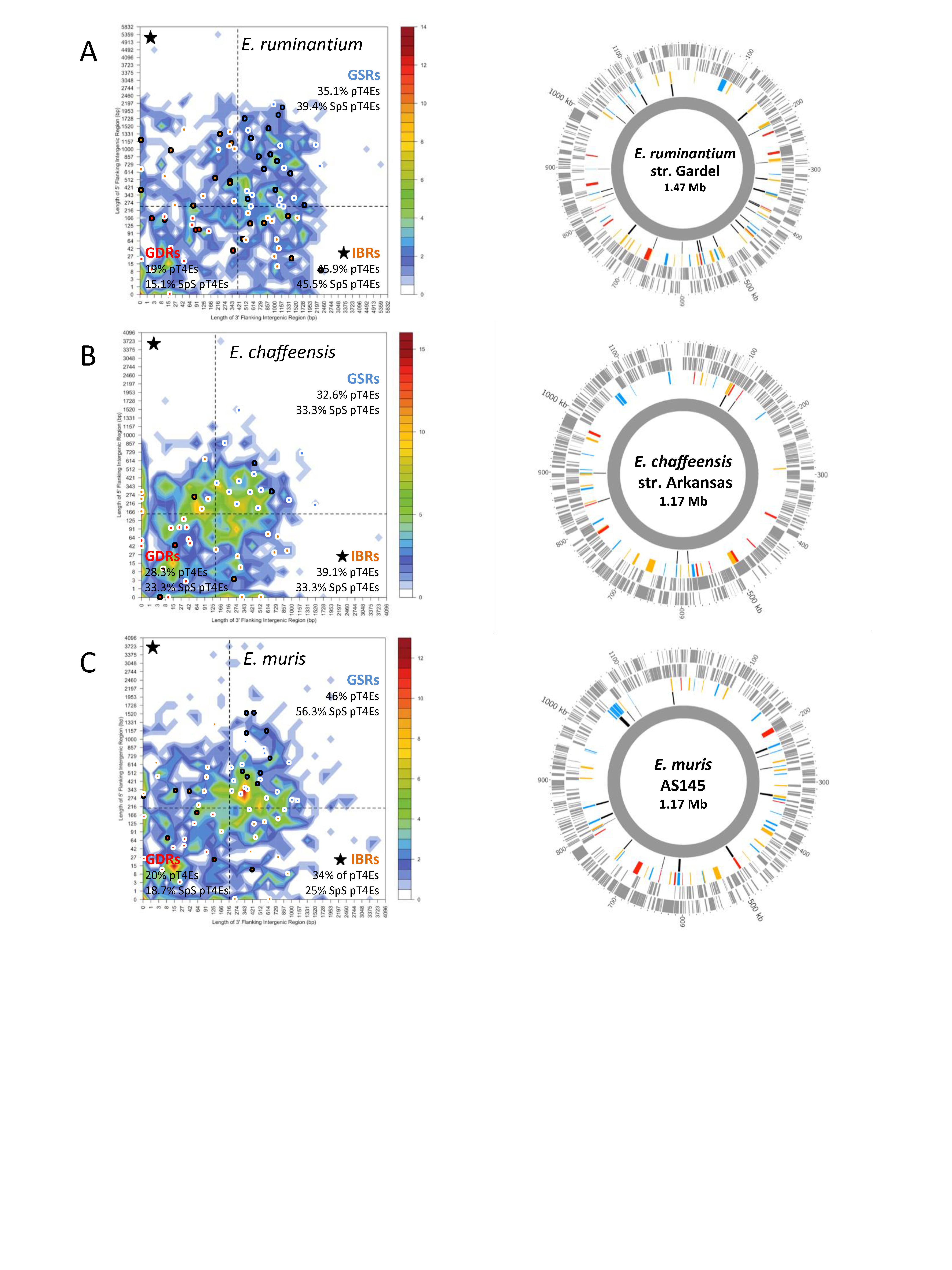

Supplement: S3 Fig — Distribution of E. ruminantium str. Gardel, E. chaffeensis str. Arkansans and E. muris AS145 genes according to the length of their flanking intergenic regions (FIRs). All the genes of each species were sorted into two-dimensional bins according to the length of their 5′ (y-axis) and 3′ (x-axis) FIR lengths. The number of genes in the bins is represented by a colour-coded density graph. Genes whose FIRs were both longer than the median length of FIRs were considered as gene-sparse region (GSR) genes. Genes whose FIRs were both below the median value were considered as gene-dense region (GDR) genes. In between (IBR) genes are genes with a long 5′ FIR and short 3′ FIR, and inversely. For E. ruminantium, E. chaffeensis and E. muris, median values are 246 bp, 156 bp and 207 bp for 5′ FIRs respectively and 405 bp, 138 bp and 219 bp for 3′ FIRs respectively. The dashed line stands for the median length of FIR and delimits the genes in GSR, GDR and IBR. Candidate effectors predicted using the S4TE 2.0 algorithm were s plotted on this distribution according to their own 3′ and 5′ FIRs. A colour was assigned to each of the three following groups: red to GDRs, orange to IBRs, and blue to GSRs. Specific pT4Es are represented with a dot outlined in black. On the right, a Circos graph shows the distribution of E. ruminantium str. Gardel, E. chaffeensis str. Arkansans and E. muris AS145 pT4Es along the genome. The colour (red, orange or blue) of each gene corresponds to their location in GDR, IBR or GSR regions respectively. (TIFF) [file pcbi.1008788.s003.tiff]

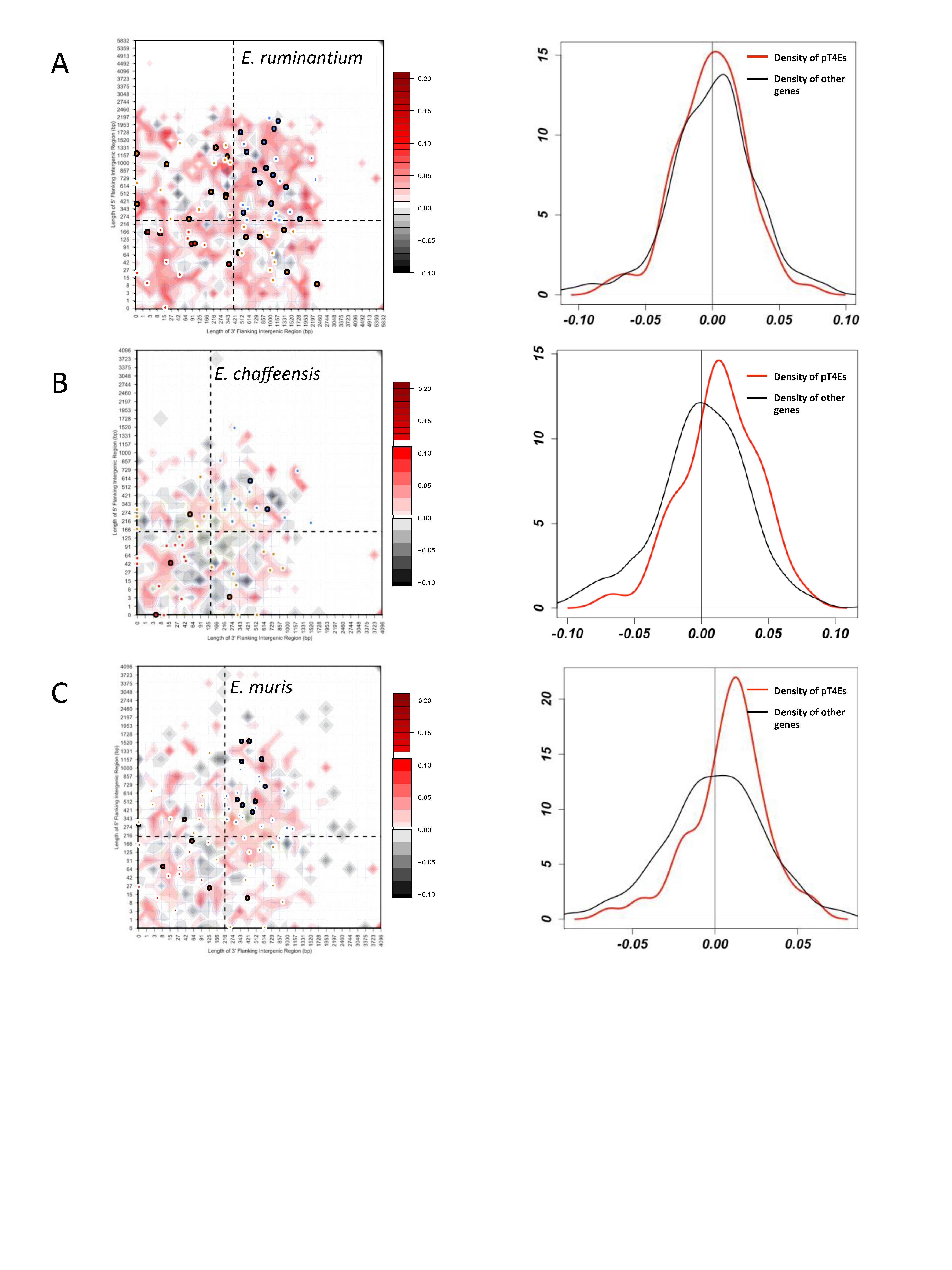

Supplement: S4 Fig — Distribution of E. ruminantium str. Gardel, E. chaffeensis str. Arkansans and E. muris AS145 genes according to the length of their flanking intergenic regions (FIRs). All the genes of each species were sorted into two-dimensional bins according to the length of their 5′ (y-axis) and 3′ (x-axis) FIRs. For each gene, the ΔGC content was calculated by subtracting the GC content of a gene by the average of GC content of all the genes. The mean of ΔGC of genes in the bins is represented by a colour-coded density graph. Genes whose FIRs were both longer than the median length of FIRs were considered as gene-sparse region (GSR) genes. Genes whose FIRs were both below the median value were considered as gene-dense region (GDR) genes. In between (IBR) genes are genes with a long 5′ FIR and short 3′ FIR, and inversely. For E. ruminantium, E. chaffeensis and E. muris, median values are 246 bp, 156 bp and 207 bp for 5′ FIRs, respectively, and 405 bp, 138 bp and 219 bp for 3′ FIRs, respectively. The dashed line showing the median length of FIR delimits the genes in GSR, GDR and IBR. A colour was assigned to each of the three following groups: red to GDRs, orange to IBRs, and blue to GSRs. Specific pT4Es are represented with a dot outlined in black. A density graph is plotted in the top right corner. The red line represents the density of pT4Es according to ΔGC content and the black line represents the density of the other genes. (TIFF) [file pcbi.1008788.s004.tiff]
